# A Multinational Case Series Describing Successful Treatment of Persistent Severe Acute Respiratory Syndrome Coronavirus 2 Infection Caused by Omicron Sublineages With Prolonged Courses of Nirmatrelvir/Ritonavir

**DOI:** 10.1093/ofid/ofad612

**Published:** 2023-12-07

**Authors:** Luke B Snell, Aimee McGreal-Bellone, Clemency Nye, Sarah Gage, Prijay Bakrania, Tom G S Williams, Emma Aarons, Alina Botgros, Samuel T Douthwaite, Patrick Mallon, Iain Milligan, Catherine Moore, Brendan O’Kelly, Jonathan Underwood, Eoghan de Barra, Gaia Nebbia

**Affiliations:** Department of Infectious Diseases, King’s College London, London, UK; Directorate of Infection, Guy’s & St Thomas’ National Health Service Foundation Trust, London, UK; Department of Infectious Diseases, Beaumont Hospital, Dublin, Ireland; Microbiology Department, Public Health Wales, Cardiff, UK; Department of Infectious Diseases, University Hospital of Wales, Cardiff, UK; Directorate of Infection, Guy’s & St Thomas’ National Health Service Foundation Trust, London, UK; Directorate of Infection, Guy’s & St Thomas’ National Health Service Foundation Trust, London, UK; Directorate of Infection, Guy’s & St Thomas’ National Health Service Foundation Trust, London, UK; Directorate of Infection, Guy’s & St Thomas’ National Health Service Foundation Trust, London, UK; Directorate of Infection, Guy’s & St Thomas’ National Health Service Foundation Trust, London, UK; Centre for Experimental Pathogen Host Research, University College Dublin, Dublin, Ireland; Directorate of Infection, Guy’s & St Thomas’ National Health Service Foundation Trust, London, UK; Wales Specialist Virology Centre, Public Health Wales, Cardiff, UK; Department of Infectious Diseases, Beaumont Hospital, Dublin, Ireland; Infectious Diseases, Our Lady of Lourdes Hospital, Drogheda, Ireland; Department of Infectious Diseases, University Hospital of Wales, Cardiff, UK; Division of Infection and Immunity, Cardiff University, Cardiff, UK; Department of Infectious Diseases, Beaumont Hospital, Dublin, Ireland; Department of International Health and Tropical Medicine, Royal College of Surgeons in Ireland, University of Medicine and Health Sciences, Ireland; Department of Infectious Diseases, King’s College London, London, UK; Directorate of Infection, Guy’s & St Thomas’ National Health Service Foundation Trust, London, UK

**Keywords:** SARS-CoV-2, COVID-19, Immunocompromise

## Abstract

The optimum treatment for persistent infection with severe acute respiratory syndrome coronavirus 2 (SARS-CoV-2) is not known. Our case series, across 5 hospitals in 3 countries, describes 11 cases where persistent SARS-CoV-2 infection was successfully treated with prolonged courses (median, 10 days [range, 10–18 days]) of nirmatrelvir/ritonavir (Paxlovid). Most cases (9/11) had hematological malignancy and 10 (10/11) had received CD20-depleting therapy. The median duration of infection was 103 days (interquartile range, 85–138 days). The majority (10/11) were hospitalized, and 7 (7/11) had severe/critical disease. All survived and 9 of 11 demonstrated viral clearance, almost half (4/9) of whom received nirmatrelvir/ritonavir as monotherapy. This case series suggests that prolonged nirmatrelvir/ritonavir has a role in treating persistent infection.

Immunocompromised individuals can suffer from persistent infection with severe acute respiratory syndrome coronavirus 2 (SARS-CoV-2) [1], the treatment of which is poorly evidenced by only case reports and case series.

The presentations, severity, and optimal treatment for persistent infection may have changed since previous reports [2] for a number of reasons. First, there is widespread existing immunity from prior infection or vaccination. Second, Omicron and its sublineages may have less intrinsic pathogenicity [3]. The treatment landscape has also changed, with newer therapies like the antiviral nirmatrelvir/ritonavir (Paxlovid) and recommendations for preemptive therapy in high-risk populations [4]. In addition, current circulating lineages of SARS-CoV-2 show resistance to neutralization by currently available therapeutic monoclonal antibodies [5].

In the new era of the SARS-CoV-2 pandemic the optimal agents and duration of treatment for persistent infection is not known. Small case series advocate for dual direct-acting antivirals (nirmatrelvir/ritonavir and remdesivir) [6–8]; others report using antivirals with plasma products [9, 10]. However these case series are small, due to the rarity of cases, featuring cases in single digits.

Our experience is to use prolonged courses of nirmatrelvir/ritonavir, so we performed a retrospective review of the treatment of persistent infection across 3 multisite healthcare institutions in 3 countries.

## METHODS

Three multisite healthcare institutions were included: (1) Guy’s & St Thomas’ National Health Service (NHS) Foundation Trust (London, England); (2) Royal College of Surgeons in Ireland Hospitals (Dublin, Ireland); and (3) University Hospital of Wales (Cardiff, Wales). Retrospective review was performed capturing all persistently infected SARS-CoV-2 cases fulfilling the following criteria: (1) continued positivity in respiratory samples for SARS-CoV-2 by polymerase chain reaction (PCR) for ≥6 weeks; (2) symptoms consistent with SARS-CoV-2 infection; (3) diagnosis of persistent infection agreed upon by infection specialists or multidisciplinary teams; (4) confirmed infection with Omicron sublineages or if no lineage available, any case diagnosed after 1 January 2022; and (5) treatment with nirmatrelvir/ritonavir. As a further exploratory analysis we also looked for cases meeting criteria but which were treated with other agents and/or were persistent for 30 days rather than 6 weeks.

Demographic information was collected including age, sex, and comorbidities using a structured data collection form. Clinical information on SARS-CoV-2 infection was retrieved including SARS-CoV-2 testing history, clinical course, severity of infection, and SARS-CoV-2 treatments. Length of infection was counted from whichever came first between (1) positive by lateral flow; (2) positive SARS-CoV-2 PCR; or (3) coronavirus disease 2019 (COVID-19) symptoms. Severity of infection was classified according to World Health Organization definitions [11]. Outcomes included symptomatic recovery and virological clearance by PCR testing on nose and throat swabs. Routine laboratory data were retrieved including immunoglobulin levels, lymphocyte subsets, and SARS-CoV-2 immunoglobulin G (IgG) status.

Informed consent was given for off-label use of nirmatrelvir/ritonavir and agreed by local drug and therapeutic committees. This study was undertaken using retrospective analysis of routinely collected clinical data, which were anonymized before analysis by the research team. As such, in the United Kingdom, formal research ethics committee review was not required as confirmed by the NHS Health Research Authority. Data from Irish sites were collected under written informed consent as part of the All Ireland Infectious Diseases Cohort Study, which was approved by the National Research Ethics Committee in Ireland.

Graphs were created in StataMP (StataCorp, College Station, Texas; Figure 1) or matlibplot [12] (Supplementary Figures 1–11). Multiple platforms were used for PCR testing and no cycle threshold correction or normalization between platforms was conducted.

**Figure 1.**
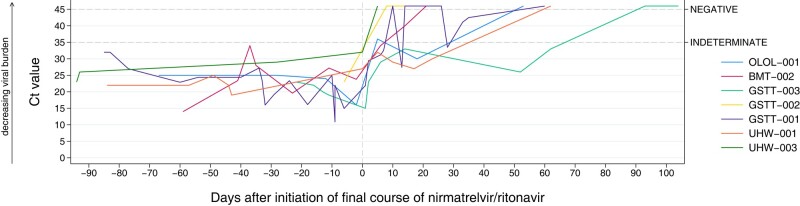
Time series for each case, displaying cycle threshold (Ct) values of SARS-CoV-2 on reverse-transcription quantitative polymerase chain reaction over time for 7 cases. Cases without follow-up testing data (n = 2) and cases where upper respiratory tract sampling was consistently negative (n = 2) are not shown. Sampling date is presented in relation to the start date of the final course of nirmatrelvir/ritonavir. Samples are shown up to 90 days prior to this final course. Samples reported as not detected are set to Ct = 46 for the purpose of graphical representation. Multiple platforms were used for testing, and no correction or normalization between platforms was conducted.

## RESULTS

Eleven cases from 5 hospitals were identified (Table 1 and Supplementary Table 1). The cases had a median age of 65 years (interquartile range [IQR], 51–77 years), with the majority (8/11 [73%]) being female.

**Table 1.** Summary Characteristics of the 11 Cases of Persistent Severe Acute Respiratory Syndrome Coronavirus 2 Infection Treated With Prolonged Nirmatrelvir/Ritonavir

Patient ID	Sex	Age	Comorbidity	CD20 Depletion	Days Infected	Maximum Respiratory Support	Severity	Outcome		Treatment Courses	Final Treatment Course		Lineage
								Clinical	Virological		Paxlovid	Combination	
OLOL-001	F	50	Hematological malignancy	Yes	72	Low-flow oxygen	Moderate	Recovered	Clear	3	10 d	Remdesivir 5 d	BQ.1.1.2
BMT-002	F	48	NMDA receptor encephalitis	Yes	76	Noninvasive ventilation	Critical	Recovered	Clear	3	18 d	Immunoglobulin	Unknown
BMT-001	F	48	Rheumatoid arthritis	Yes	103	High-flow oxygen	Severe	Recovered	LTFU	1	10 d	Immunoglobulin	Unknown
GSTT-005	F	77	Hematological malignancy	Yes	76	High-flow oxygen	Severe	Recovered	LTFU	2	10 d	Sotrovimab 1000 mg	BQ.1.1
GSTT-004	F	77	Hematological malignancy	Yes	203	Nonhospitalized	Mild	Recovered	Clear	1	10 d	Sotrovimab 1000 mg	BQ.1.1
GSTT-003	F	77	Hematological malignancy	Yes	101	High-flow oxygen	Severe	Recovered	Clear	2	10 d	Remdesivir 10 d + sotrovimab 1000 mg	BQ.1.1.3
GSTT-002	M	55	Hematological malignancy	Yes	236	Hospitalized, no oxygen	Mild	Recovered	Clear	1	10 d	Nil	BA.2.9
GSTT-001	M	61	Hematological malignancy	Yes	137	Invasive ventilation	Critical	Recovered	Clear	4	10 d	Remdesivir 10 d	BA.2.3
UHW-001	M	74	Hematological malignancy; rheumatoid arthritis	Yes	134	CPAP	Critical	Recovered	Clear	5	10 d	Nil	BA.2
UHW-002	F	80	Hematological malignancy	Yes	138	CPAP	Critical	Recovered	Clear	3	14 d	Nil	BL.1
UHW-003	F	68	Hematological malignancy	Nil	94	Low-flow oxygen	Moderate	Recovered	Clear	2	10 d	Nil	BQ.1.1.8

Abbreviations: CPAP, continuous positive airway pressure; d, days; F, female; LTFU, lost to follow-up; M, male; NMDA, *N*-methyl-d-aspartate receptor.

Of the comorbidities and associated immunosuppression, 9 individuals had hematological malignancy and 2 individuals had rheumatoid arthritis, and there was 1 case of *N*-methyl-d-aspartate receptor (NMDA) receptor encephalitis. Ten (10/11) patients were treated with anti-CD20 biological therapy, mostly (9/10) within the last 6 months. All patients had received ≥3 doses of COVID-19 vaccine prior to infection.

The median length of SARS-CoV-2 infection was 103 days (IQR, 85–138 days). Where lineage information was available (9/11), all were sublineages of Omicron BA.2 or BA.5. All were symptomatic with respiratory symptoms and all had radiological pulmonary changes consistent with SARS-CoV-2 infection. Maximal disease severity included 10 of 11 requiring hospital admission, with 2 of 11 having mild disease, 2 of 11 moderate disease, and 7 of 11 severe or critical disease. For those 7 with severe or critical disease, 3 received high-flow oxygen, 2 continuous positive airway pressure, 1 noninvasive ventilation, and 1 invasive ventilation.

We reviewed available immunological results taken during infection. Ten (10/11) had totaI IgG tested, with 2 (2/10) having undetectable IgG and a further 6 (6/10) having IgG below the limit of normal. Ten (10/11) had SARS-CoV-2 IgG tested and 8 (80%) were reported as not detected. Of 6 (6/11) individuals who had lymphocyte subsets tested, 5 had low circulating B cells and 2 had CD4 count <150 cells/μL.

Cases received a median of 2 treatment courses (range, 1–5; see Supplementary Table 1 for full treatment information). The last treatment in all (11/11) cases including a prolonged treatment course of nirmatrelvir/ritonavir (median, 10 days [range, 10–18 days]). The cycle threshold values of SARS-CoV-2 in relation to the final treatment courses of prolonged nirmatrelvir/ritonavir are shown in Figure 1, with a detailed clinical course for each case in Supplementary Figures 1–11.

In combination with nirmatrelvir/ritonavir, 7 (7/11) received an additional agent: 2 cases received double-dose (1000 mg) sotrovimab, 2 cases received immunoglobulin, 2 cases received remdesivir, and 1 case received a prolonged course of remdesivir and double dose of sotrovimab. All but the mild cases (9/11) received steroids, and less than half (3/7) of severe/critical disease received interleukin 6 blockade.

After the final treatment course, 9 (9/11) cases cleared infection, and 2 (2/11) had symptomatic recovery and resolution of oxygen requirement but were lost to follow-up testing after discharge from local hospitals. There were no deaths. Four of the patients with demonstrated virological clearance (4/9) received nirmatrelvir/ritonavir as monotherapy.

As an additional exploratory analysis, we looked for patients with shorter durations of persistence (30 days rather than 6 weeks) and/or were treated with other agents than nirmatrelvir/ritonavir (Supplementary Table 2). Of 4 patients, all died: 2 (2/4) from COVID-19 before 6 weeks of infection duration, and 2 (2/4) from comorbid conditions after 6 weeks of infection. One (1/4) was judged to have cleared infection prior to death, after receiving 10 days of remdesivir.

## DISCUSSION

The risk of developing persistent SARS-CoV-2 infection remains unclear. However, our case series shows persistent infection can still be associated with significant morbidity in clinically extremely vulnerable individuals. This is despite our cohort all receiving ≥3 vaccine doses and reports that Omicron may be associated with reduced severity at a population level [3].

Our experience suggests that prolonged courses of nirmatrelvir/ritonavir lasting 10 days may be effective in treatment and clearance of persistent SARS-CoV-2 infection even as monotherapy. Previous case studies have suggested that dual therapy may be required with either another direct-acting antiviral [6, 8] or with polyclonal antibody therapy [9, 10]. From these series, only 2 patients were treated with prolonged nirmatrelvir/ritonavir as monotherapy [6, 8]. The optimal duration of nirmatrelvir/ritonavir is not known, with 1 case in this series receiving a treatment course of 18 days. Two patients also cleared persistent infection after repeat treatment with nirmatrelvir/ritonavir, suggesting that repeat treatment courses may be effective even after initial treatment courses fail to clear infection.

Due to the nature of this study we could not systematically ascertain the occurrence of adverse effects; however, 1 case developed thrombocytopenia, which was attributed to nirmatrelvir/ritonavir. This adverse effect is not listed in the summary of product characteristics for nirmatrelvir/ritonavir [13] but is a recognized, common side effect of ritonavir [14]. The safety of 10 days of nirmatrelvir/ritonavir has been studied in clinical trials [15], with the safety profile remaining “generally consistent” when used for either 5 or 10 days [16]; however, peer-reviewed results are awaited.

Notably, the vast majority of the patients described here were treated with B-cell–depleting therapies prior to developing persistent SARS-CoV-2 infection, a risk factor seen in prior cohorts earlier in the pandemic [2]. This immunodeficiency is reflected in the majority with undetectable SARS-CoV-2 IgG.

The use of concomitant antibody therapy (either sotrovimab or intravenous immunoglobulin) formed a part of the final treatment course in a large proportion (5/11) of cases. The practice of our 3 centers is to consider antibody therapy in cases with undetectable SARS-CoV-2 IgG. These agents offer a strategy for combination therapy with nirmatrelvir/ritonavir that does not require lengthy admissions, as would be the case if remdesivir was given as a second agent [6, 8, 10].

Three patients received sotrovimab monoclonal antibody therapy as a component of their final treatment course. Sotrovimab has reduced ability to neutralize recently circulating Omicron sublineages [17], and the only randomized controlled trial analyzing efficacy of sotrovimab in hospitalized patients failed to show effect on recovery [18]. Despite this lack of evidence for neutralization or efficacy, the continued usefulness of sotrovimab has been proposed [19, 20] and it may exert therapeutic effects by alternate mechanisms, such as antibody-dependent cellular toxicity as seen in animal models [21]. Safety of doses up to 2000 mg have been previously studied [22]. In contrast, intravenous immunoglobulin likely contains polyclonal antibody against SARS-CoV-2, which can neutralize emerging variants [10].

Some of our successfully treated patients with viral clearance showed continued PCR positivity, albeit at low levels, even after the final treatment course. This was interpreted as nonviable viral RNA shed over the next few weeks. We suggest that continued improvement of the patient clinically and radiologically should be used alongside PCR testing to guide both treatment duration and treatment outcome. This is consistent with our understanding of infection and testing seen in acute infection, where RNA positivity can be seen up to 60 days and is not interpreted to indicate ongoing infection [23].

Whether additional agents in addition to nirmatrelvir/ritonavir offers clinical benefit is unclear, for instance in those with more severe disease. Treatment decisions across centers and between patients were heterogeneous and did not follow a set protocol. A multisite, randomized clinical trial is needed to provide evidence for which agents, in which combination, provide effective treatment for persistent SARS-CoV-2 infection in immunocompromised individuals. Our data suggest that combination therapy may not be necessary, as a similar number of patients who cleared infection received only monotherapy with prolonged nirmatrelvir/ritonavir as their final treatment course. This case series suggests a role for prolonged nirmatrelvir/ritonavir in the treatment of persistent infection.

## Supplementary Material

ofad612_Supplementary_Data

## Supplementary Data

Supplementary materials are available at *Open Forum Infectious Diseases* online. Consisting of data provided by the authors to benefit the reader, the posted materials are not copyedited and are the sole responsibility of the authors, so questions or comments should be addressed to the corresponding author.

## Notes

***Patient consent statement.*** For the UK sites, formal Research Ethics Committee review was not required as confirmed by the NHS Health Research Authority. For Irish sites, written informed consent was obtained.

***Financial support***. L. B. S., J. U., and G. N. receive funding from the Medical Research Council (L. B. S.: MR/W025140/1 and MC_PC_19041; J. U.: MR/T023791/1; G. N.: MR/T005416/1 and MC_PC_19041).
