# Supplementary material for: A Multinational Case Series Describing Successful Treatment of Persistent Severe Acute Respiratory Syndrome Coronavirus 2 Infection Caused by Omicron Sublineages With Prolonged Courses of Nirmatrelvir/Ritonavir
Source: Open Forum Infect Dis. 2023 Dec 7;11(1):ofad612. doi: 10.1093/ofid/ofad612 (PMC10807981; doi:10.1093/ofid/ofad612)
Supplement: ofad612_Supplementary_Data [file ofad612_supplementary_data.zip › Supplementary Figures.pdf]

Supplementary Figures

Supplementary Figure 1. Detailed clinical course for patient GSTT-001
Supplementary Figure 2. Detailed clinical course for patient GSTT-002
Supplementary Figure 3. Detailed clinical course for patient GSTT-003
Supplementary Figure 4. Detailed clinical course for patient GSTT-004
Supplementary Figure 5. Detailed clinical course for patient GSTT-005
Supplementary Figure 6. Detailed clinical course for patient UHW-001
Supplementary Figure 7. Detailed clinical course for patient UHW-002
Supplementary Figure 8. Detailed clinical course for patient UHW-003
Supplementary Figure 9. Detailed clinical course for patient BMT-001
Supplementary Figure 10. Detailed clinical course for patient BMT-002
Supplementary Figure 11. Detailed clinical course for patient OLOL-001

Legend for Supplementary Figures

Timeline of chronic COVID-19 illness, viral load and treatments for each case.

Panel A shows serial CT imaging in chronological order, where consent obtained.. Panel B shows positive LFT tests and Ct values of SARS-CoV-2 PCR results, with a circle representing a Nose and Throat Swab and triangle representing a Broncho-alveolar lavage sample. Black markers represent positive results, grey markers are indeterminate, and open markers are negative results. The background colour of panel B shows the patient's maximum level of care during that admission: white = not admitted and not on oxygen, grey = not admitted and on ambulatory oxygen, blue = admitted to a ward, orange = admitted to ITU, red = intubated. Panel C is a timeline of antiviral and immunomodulatory treatments. For corticosteroid treatments the dose is represented by a grey bar, with thickness denoting relative dose.

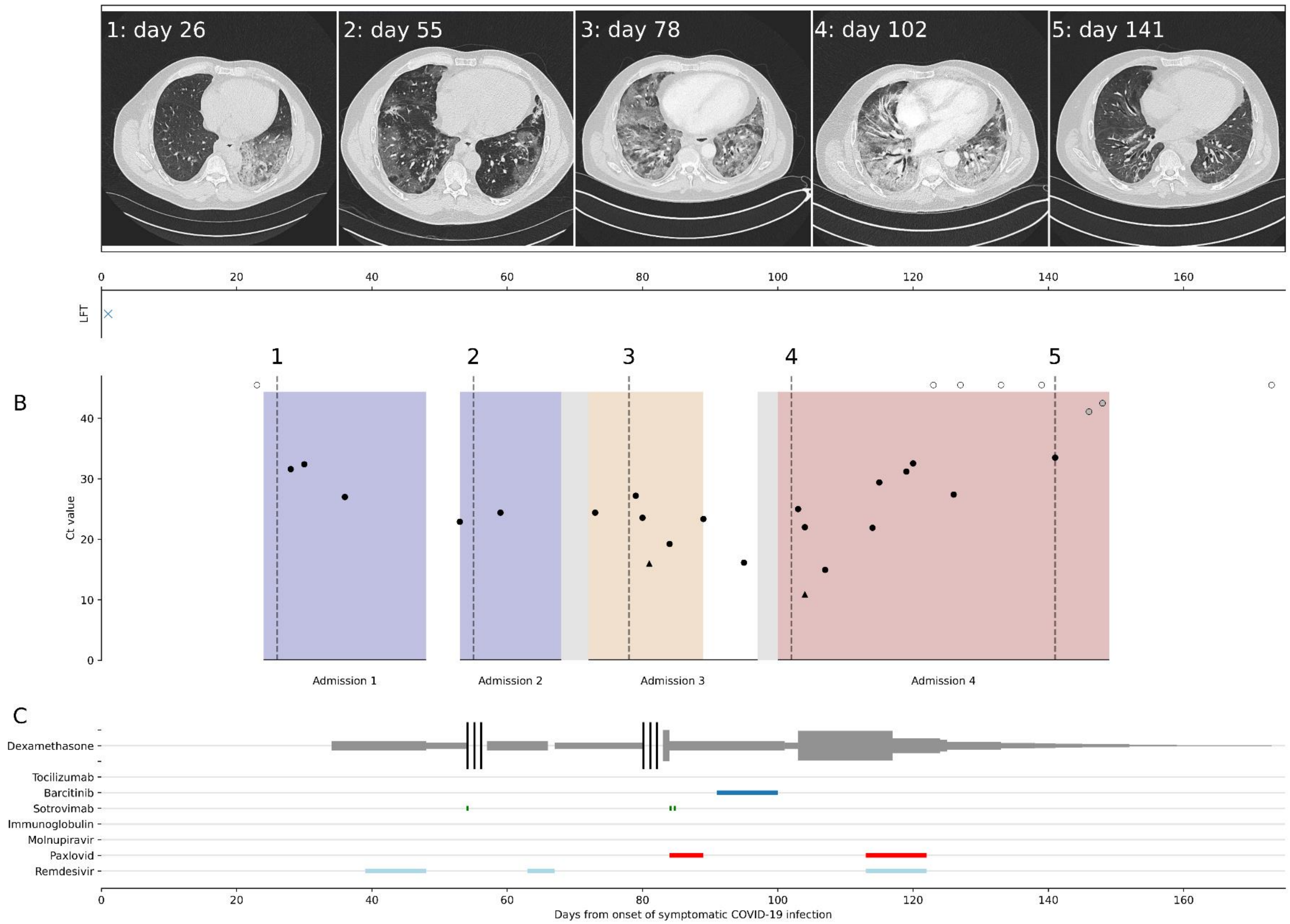

Supplementary Figure 1. Detailed clinical course for patient GSTT-001

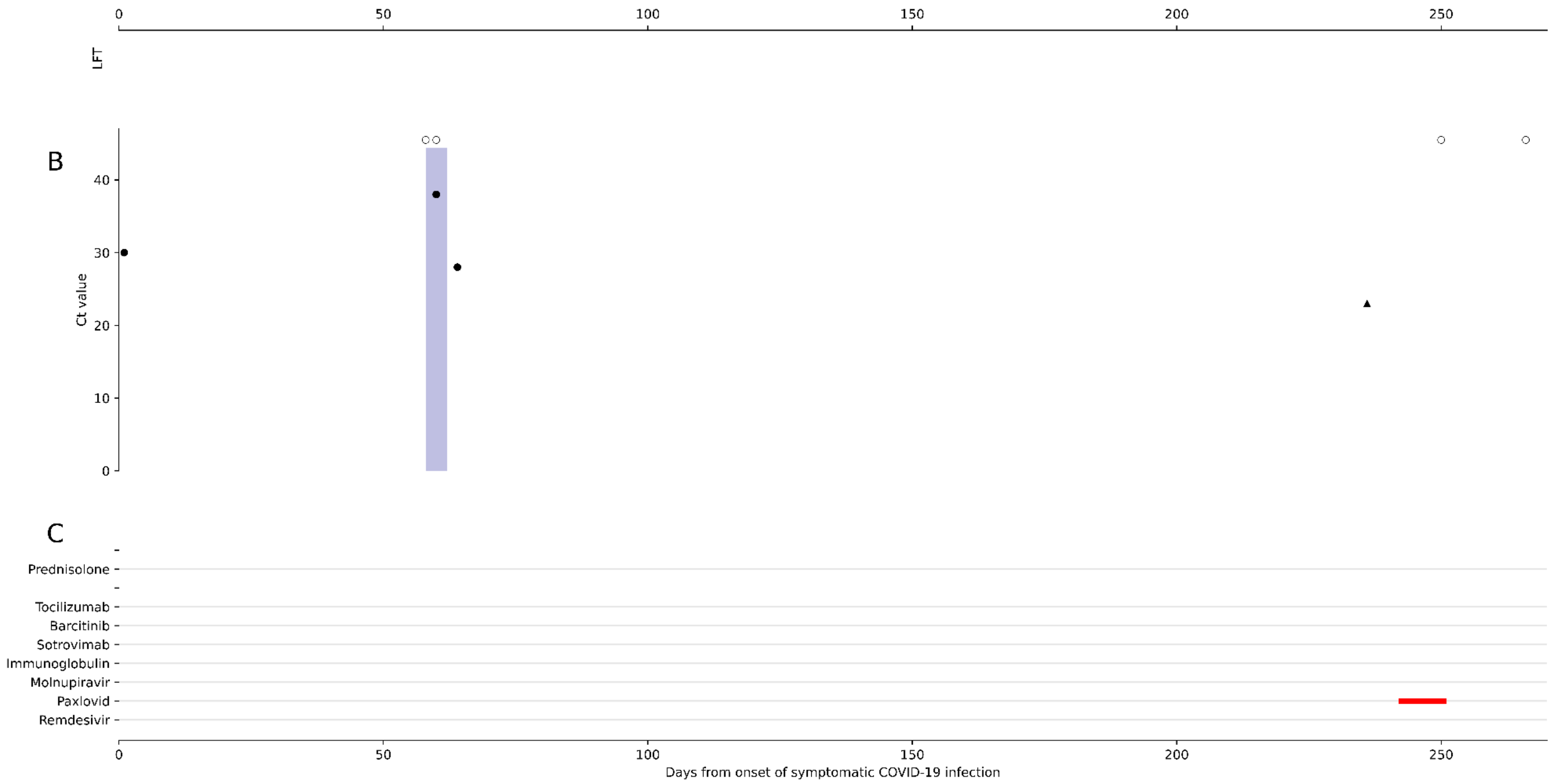

Supplementary Figure 2. Detailed clinical course for patient GSTT-002

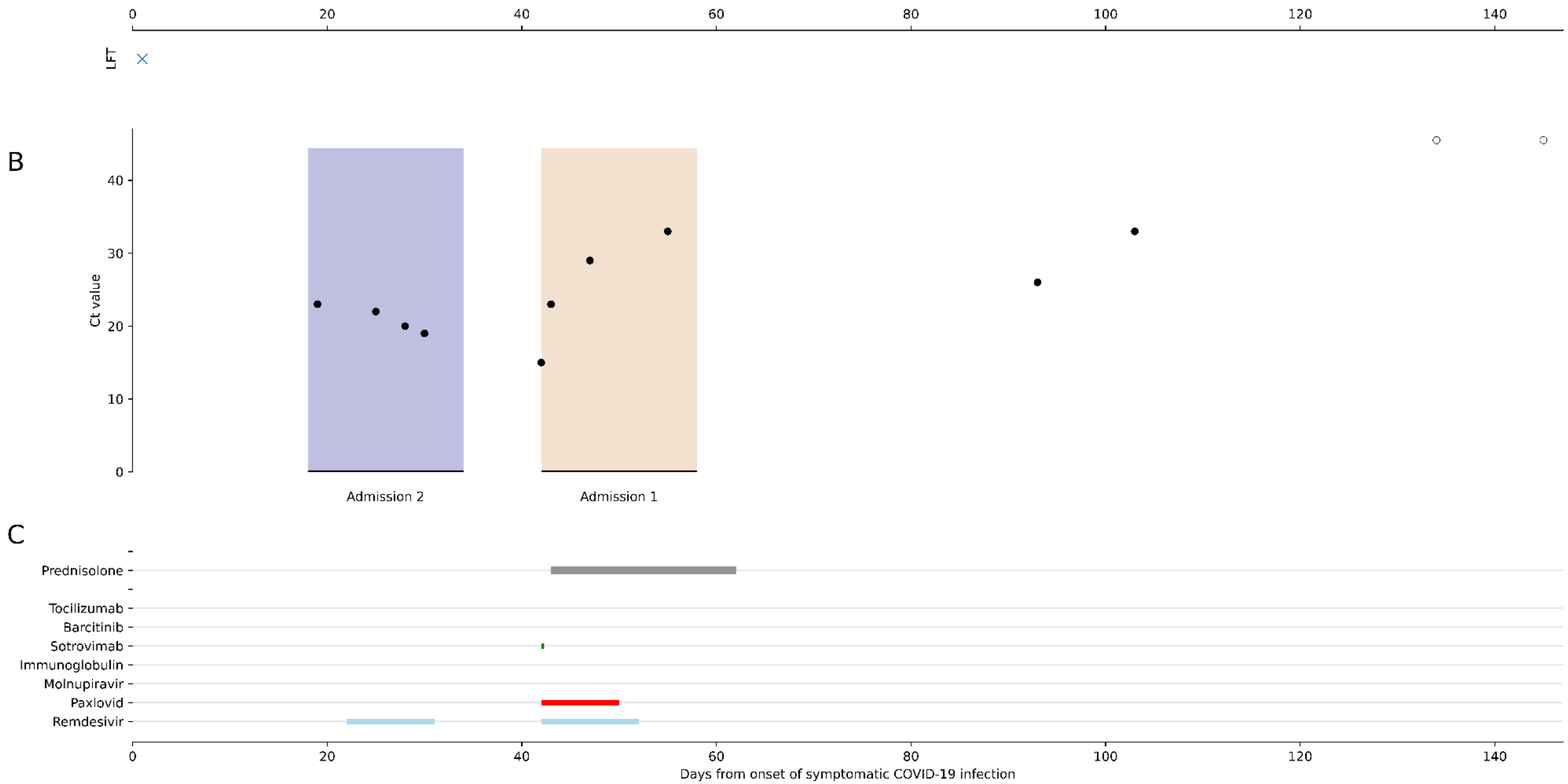

Supplementary Figure 3. Detailed clinical course for patient GSTT-003

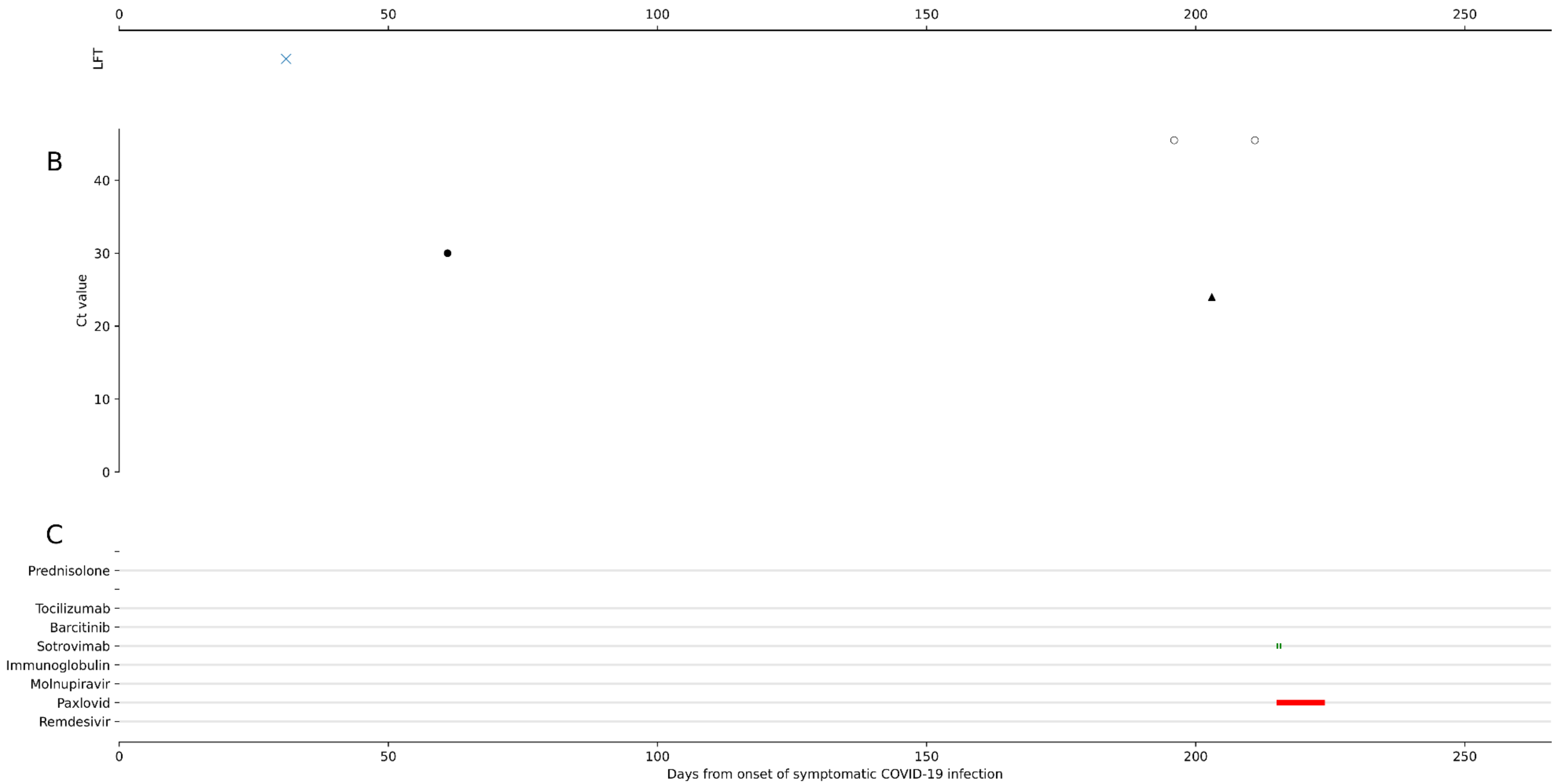

Supplementary Figure 4. Detailed clinical course for patient GSTT-004

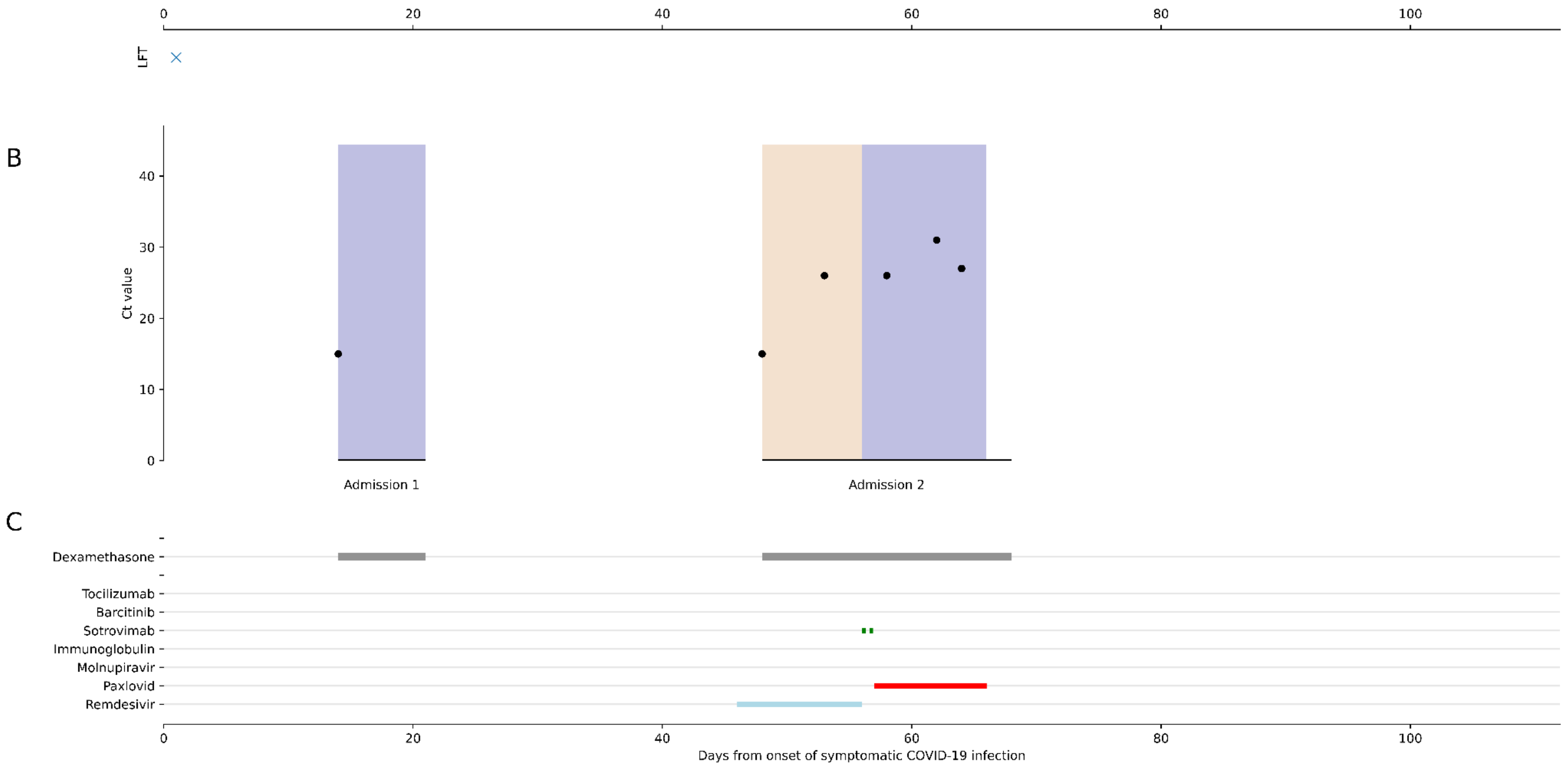

<u>Supplementary Figure 5</u>. Detailed clinical course for patient GSTT-005

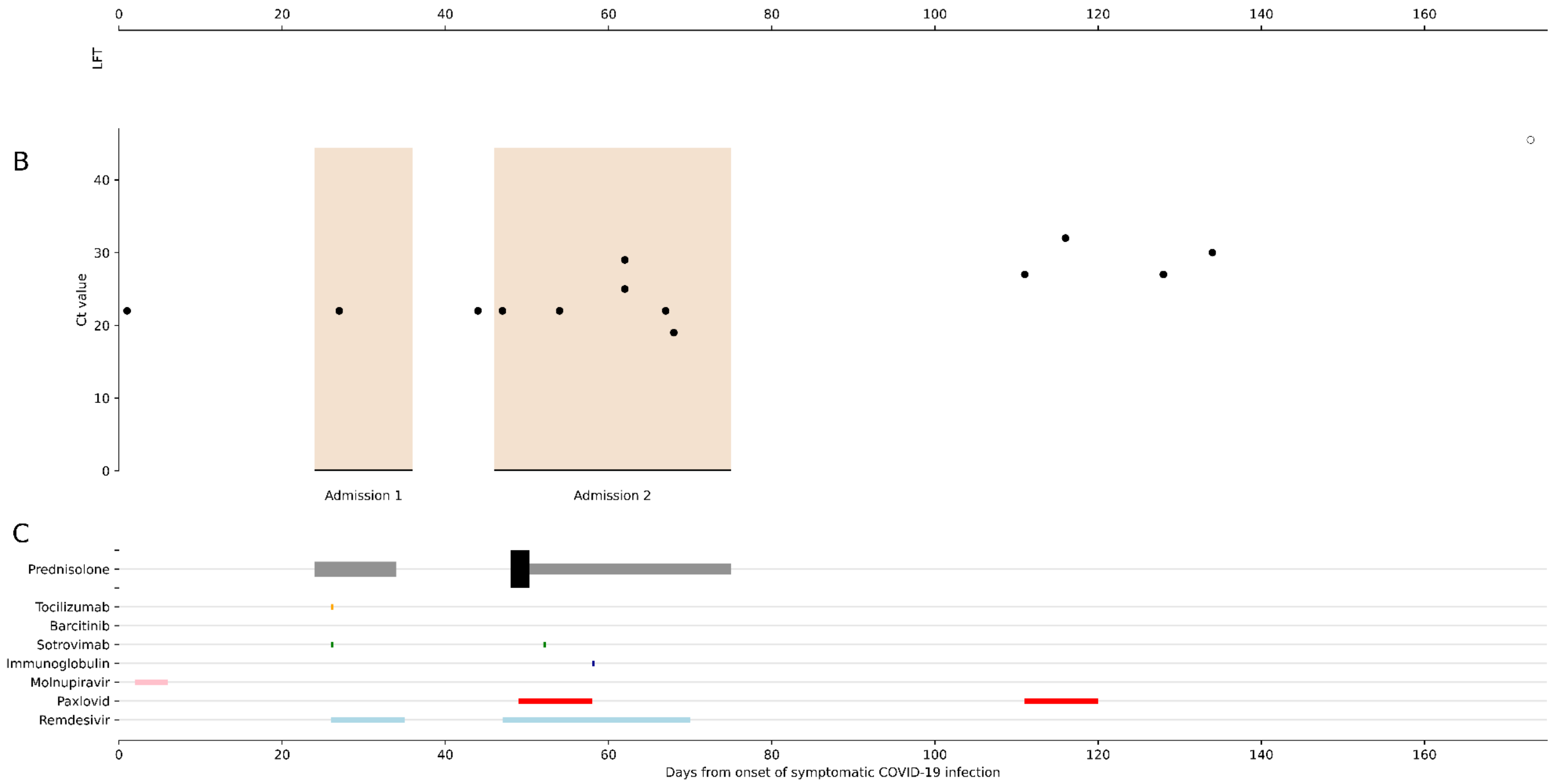

Supplementary Figure 6. Detailed clinical course for patient UHW-001

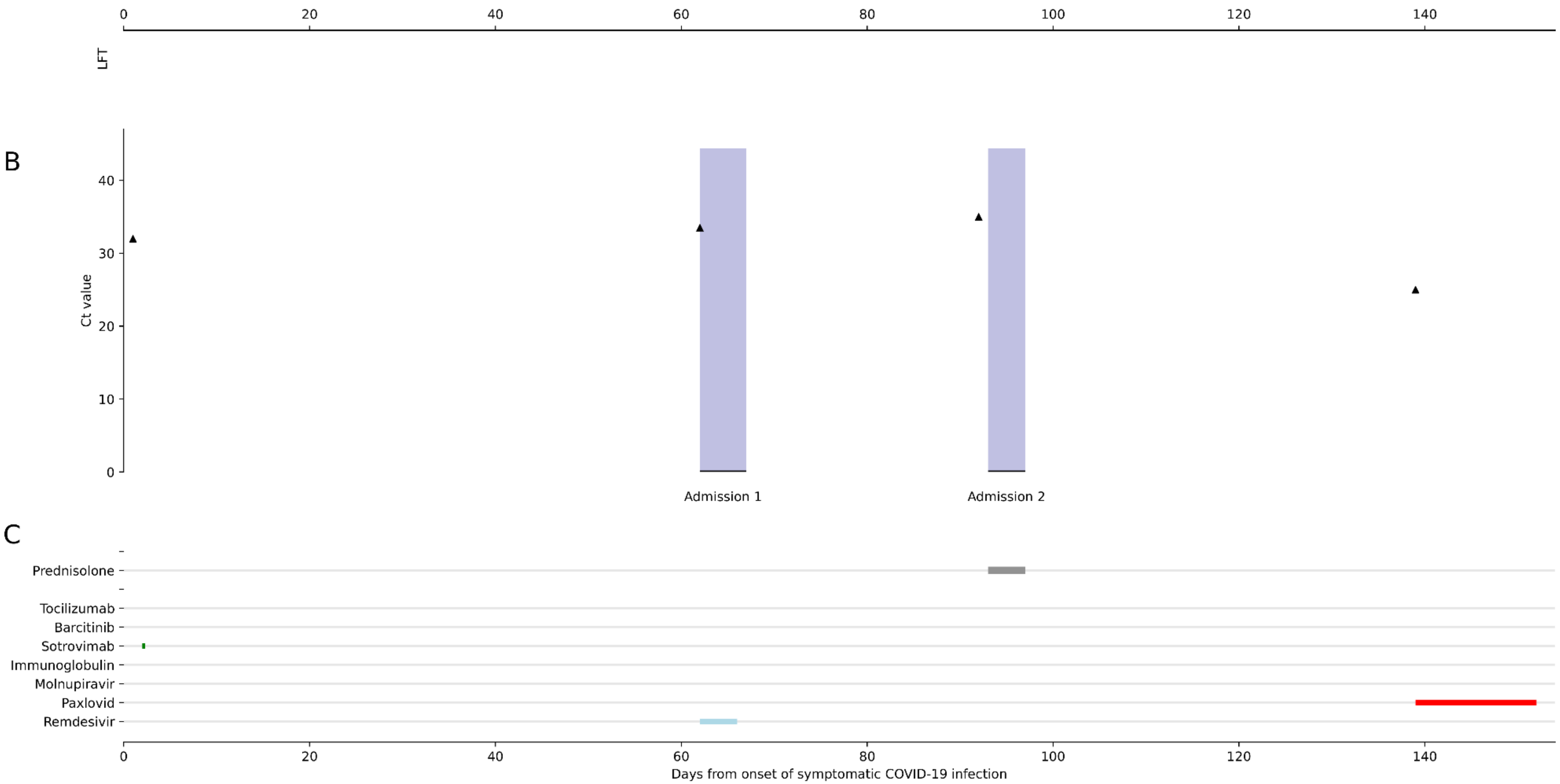

Supplementary Figure 7. Detailed clinical course for patient UHW-002

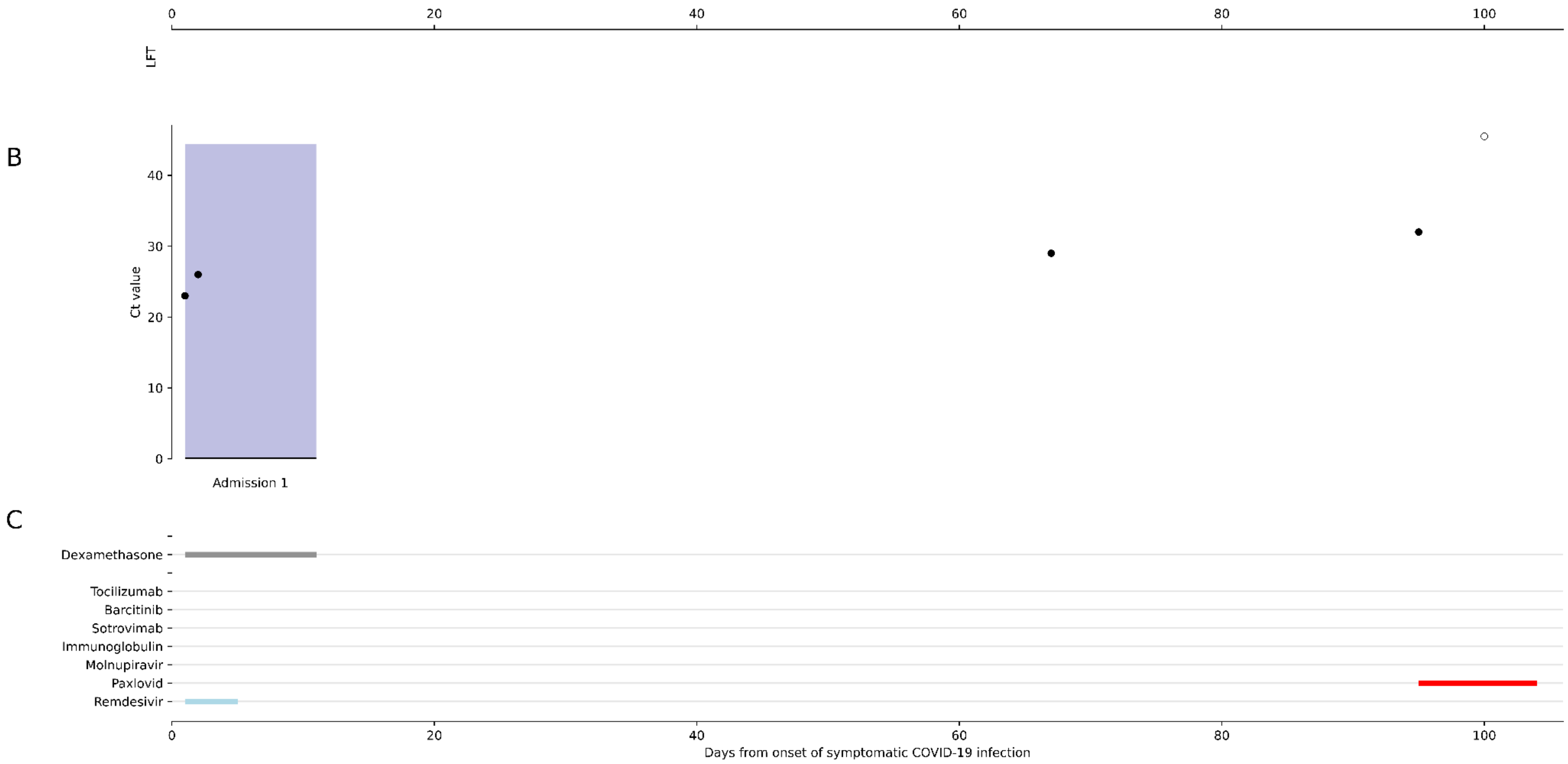

Supplementary Figure 8. Detailed clinical course for patient UHW-003

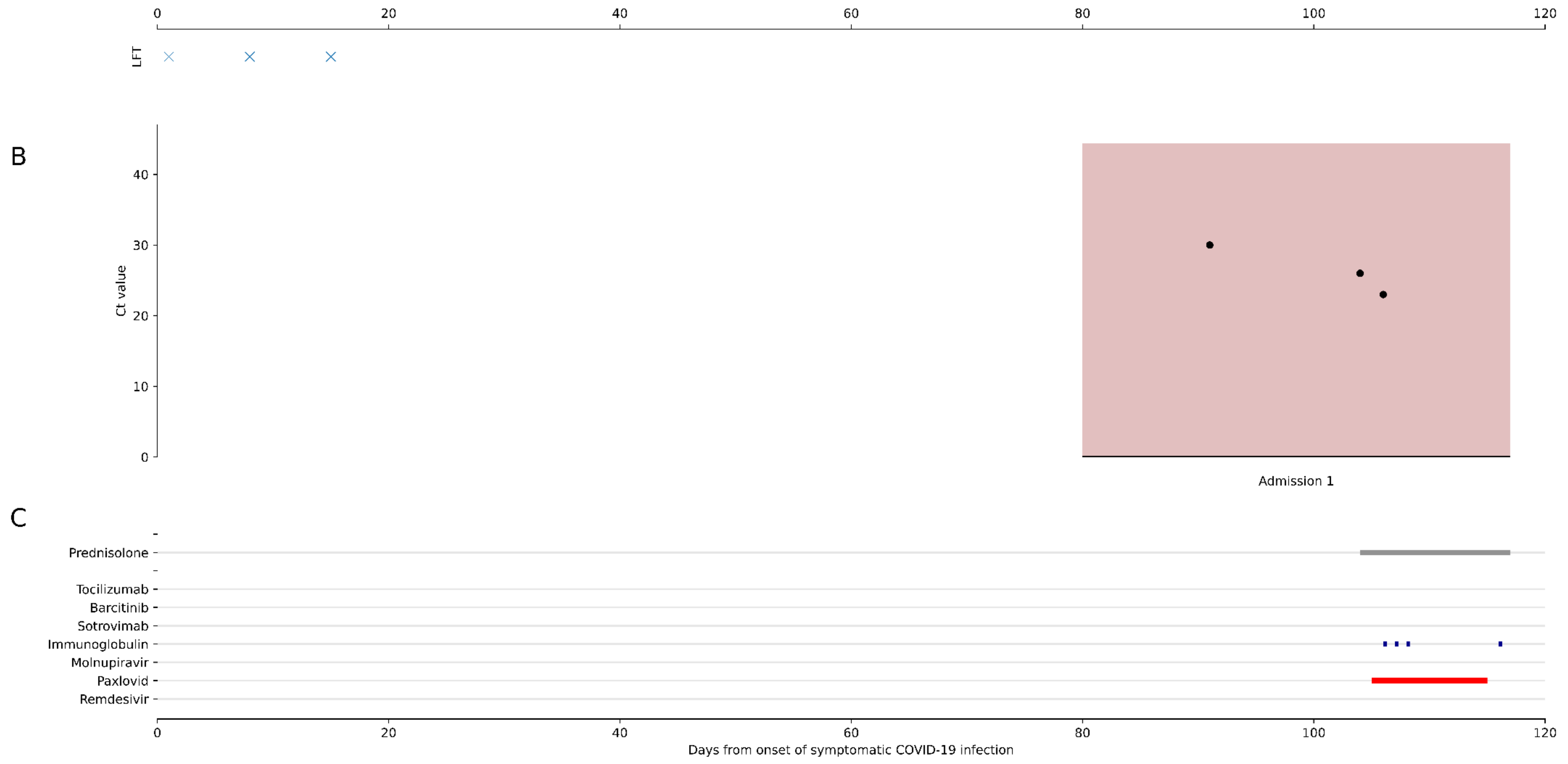

Supplementary Figure 9. Detailed clinical course for patient BMT-001

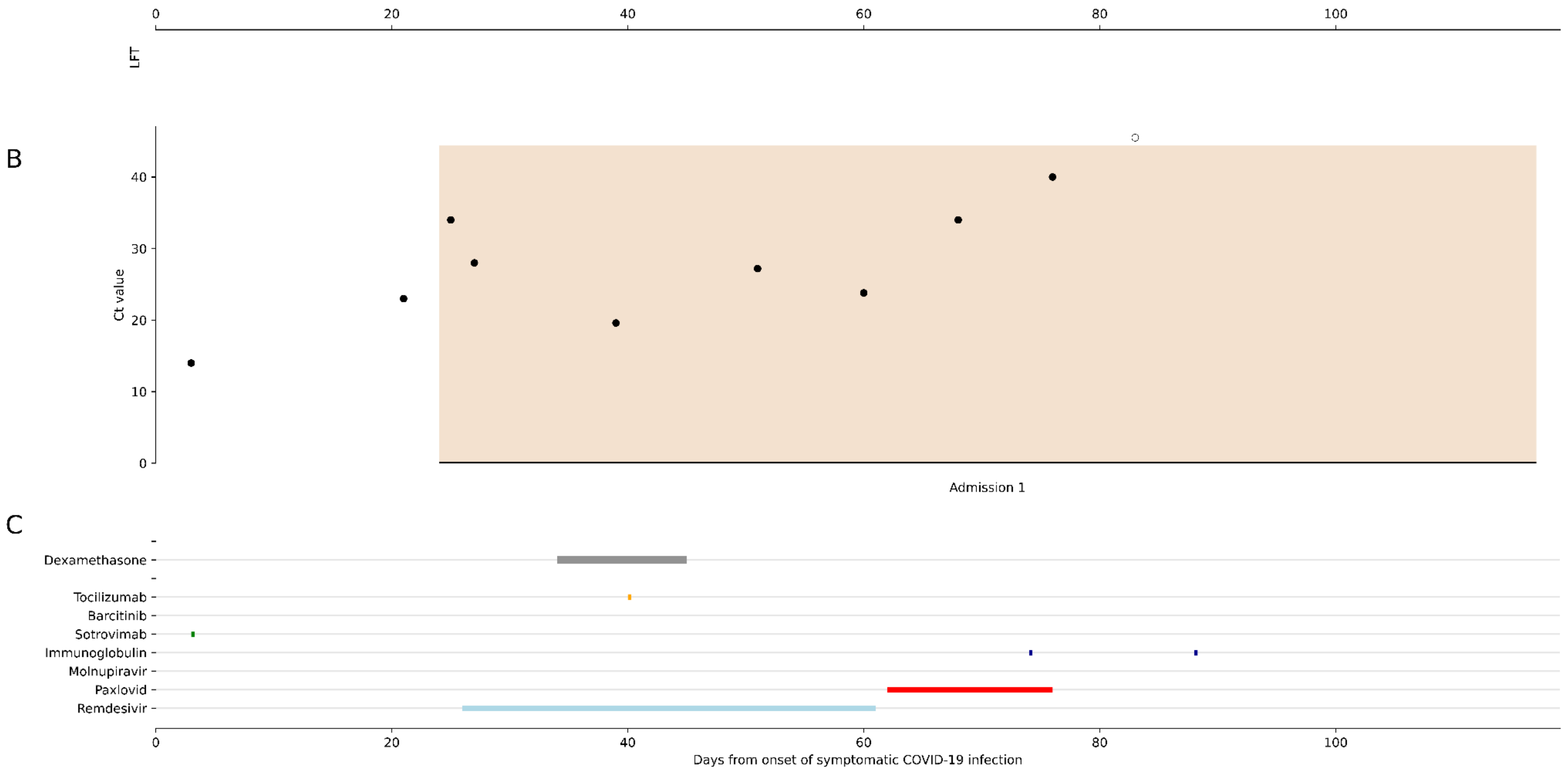

Supplementary Figure 10. Detailed clinical course for patient BMT-002

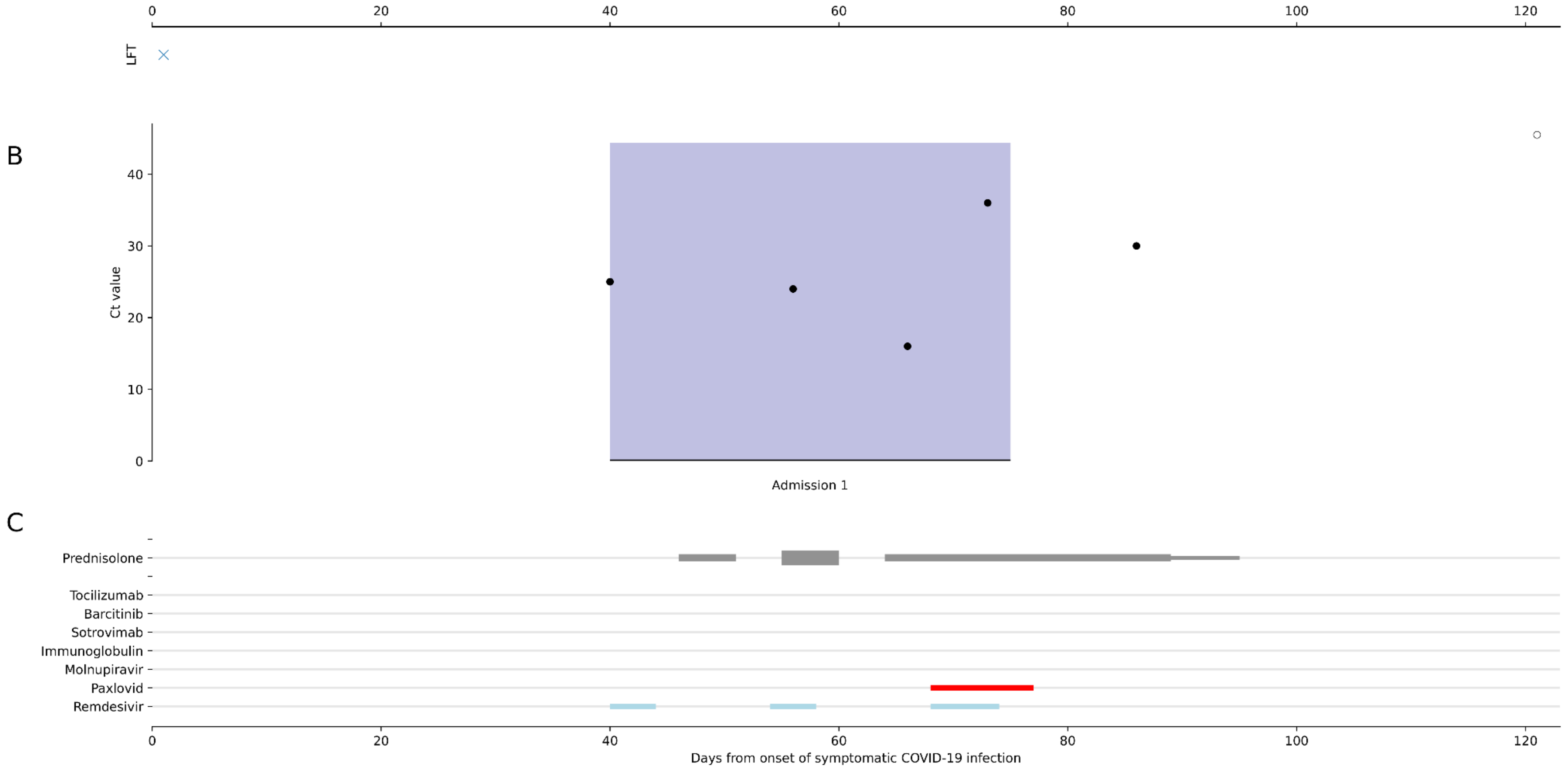

Supplementary Figure 11. Detailed clinical course for patient OLOL-001
